# Supplementary material for: Lactose Content and Selected Quality Parameters of Sheep Milk Fermented Beverages during Storage
Source: Animals (Basel). 2022 Nov 10;12(22):3105. doi: 10.3390/ani12223105 (PMC9686720; doi:10.3390/ani12223105)
Supplement: Supplementary file 1 [file animals-12-03105-s001.zip › animals-1962295-supplementary.pdf]

Figure S1: Method of lactose content determination in sheep milk fermented beverages - the individual steps of the analytical procedure

The lactose content of the products was determined in accordance with the method described in Polish Standard PN-68/A-86122 “Milk: Research methods”. This is the reference method used to evaluate milk samples and dairy products approved by the Polish Minister of Agriculture and Rural Development in the Regulation of 12 December 2002 (Dz.U.2002.230.1931)

This method involves determining the amount of halogen reduced by the reaction of lactose in the sample with chloramine T and potassium iodide. The indirect determination involves the titration of the previously deproteinized sample, together with the reagents with a 0.04 N sodium thiosulfate solution.

The reagents required are:

- tungsten reagent
- 10% solution of potassium iodide
- 0.57% solution of chloramine T
- 2 M hydrochloric acid solution
- 1% starch solution
- 0.04 M sodium thiosulfate solution.

All reagents used for the determination were prepared immediately before the start of the study, according to a recipe of our devising:

tungsten reagent =		7 g sodium tungstate<br>870 cm^3 distilled water<br>0.1 cm^3 85% orthophosphoric acid solution, 70
	+	cm^3 1 M sulfuric acid solution
10% potassium iodide solution =		50 g potassium iodide
	+	450 cm^3 of distilled water
0.57% solution of chloramine T =		6 g 95% chloramine T solution
	+	994 cm^3 distilled water
2M hydrochloric acid solution =		100 cm^3 3M hydrochloric acid solution
	+	50 cm^3 of distilled water
1% starch solution =		1 g starch
	+	99 cm^3 of distilled water
0.04 M sodium thiosulfate solution =		9.93 g sodium thiosulphate pentahydrate,
	+	990.07 cm^3 distilled water

Our analytical procedure for determining the lactose content in the fermented milk beverages is as follows:

Measuring out 10 cm^3 of the product to be tested
using a 10 cm^3 graduated cylinder

↓

quantitative transfer to a 100 cm^3
volumetric flask

↓

determination of sample mass
using an analytical scales with a
measurement accuracy of 0.01 g

↓

addition of 25 cm^3 distilled water

↓

addition of 40 cm^3 of tungsten reagent

↓

mixing

↓

addition of distilled water to make up a volume of 100 cm^3

↓

mixing

↓

Putting aside for ten minutes to allow precipitation of protein

↓

filtration into a conical flask

↓

transferal of three aliquots of 10 cm^3 each clear filtrate
to three conical flasks with polished stoppers, of capacity 300 cm^3.
A blank sample was prepared at the same time.

↓	↓	↓	↓
10 cm^3 filtrate	10 cm^3 filtrate	10 cm^3 filtrate	10 cm^3 distilled water
↓	↓	↓	↓

addition of 5 cm^3 potassium iodide

↓

addition of 20 cm^3 chloramine T

↓

mixing

↓

setting aside in a dark, dry place for 1.5 hours

↓

addition of 5 cm^3 hydrochloric acid

↓

mixing

↓

addition of three drops of starch solution

↓

titration with sodium thiosulphate until
the starch's blue color is lost
